# The promise and challenges of combination therapies with antibody-drug conjugates in solid tumors

**DOI:** 10.1186/s13045-023-01509-2

**Published:** 2024-01-04

**Authors:** Qing Wei, Peijing Li, Teng Yang, Jiayu Zhu, Lu Sun, Ziwen Zhang, Lu Wang, Xuefei Tian, Jiahui Chen, Can Hu, Junli Xue, Letao Ma, Takaya Shimura, Jianmin Fang, Jieer Ying, Peng Guo, Xiangdong Cheng

**Affiliations:** 1 Department of Medical Oncology, Zhejiang Cancer Hospital, Hangzhou, China; 2 Hangzhou Institute of Medicine (HIM), Chinese Academy of Sciences, Hangzhou, China; 3 Key Laboratory of Prevention, Diagnosis and Therapy of Upper Gastrointestinal Cancer of Zhejiang Province, Hangzhou, China; 4 Department of Radiation Oncology, Zhejiang Cancer Hospital, Key Laboratory of Head and Neck Cancer Translational Research of Zhejiang Province, Hangzhou, China; 5 College of Pharmaceutical Science, Zhejiang University of Technology, Hangzhou, China; 6 Zhejiang Chinese Medical University, Hangzhou, China; 7 Department of Gynecologic Oncology, Zhejiang Cancer Hospital, Hangzhou, China; 8 Department of Radiation Oncology, Second Affiliated Hospital, Zhejiang University School of Medicine, Zhejiang University, Hangzhou, China; 9 Shanghai Institute of Materia Medica, University of Chinese Academy of Sciences, Shanghai, China; 10 College of Molecular Medicine, Hangzhou Institute for Advanced Study (HIAS), University of Chinese Academy of Sciences, Hangzhou, China; 11 Department of Gastric Surgery, Zhejiang Cancer Hospital, Hangzhou, China; 12 Department of Oncology, Shanghai East Hospital, School of Medicine, Tongji University, Shanghai, China; 13 Department of Gastroenterology and Metabolism, Nagoya City University Graduate School of Medical Sciences, Nagoya, Japan; 14 School of Life Science and Technology, Tongji University, Shanghai, China

**Keywords:** Antibody-drug conjugate, Solid tumor, Combination therapy, Immunotherapy, Targeted therapy

## Abstract

Antibody-drug conjugates (ADCs) represent an important class of cancer therapies that have revolutionized the treatment paradigm of solid tumors. To date, many ongoing studies of ADC combinations with a variety of anticancer drugs, encompassing chemotherapy, molecularly targeted agents, and immunotherapy, are being rigorously conducted in both preclinical studies and clinical trial settings. Nevertheless, combination therapy does not always guarantee a synergistic or additive effect and may entail overlapping toxicity risks. Therefore, understanding the current status and underlying mechanisms of ADC combination therapy is urgently required. This comprehensive review analyzes existing evidence concerning the additive or synergistic effect of ADCs with other classes of oncology medicines. Here, we discuss the biological mechanisms of different ADC combination therapy strategies, provide prominent examples, and assess their benefits and challenges. Finally, we discuss future opportunities for ADC combination therapy in clinical practice.

## Background

**Fig. 1:**
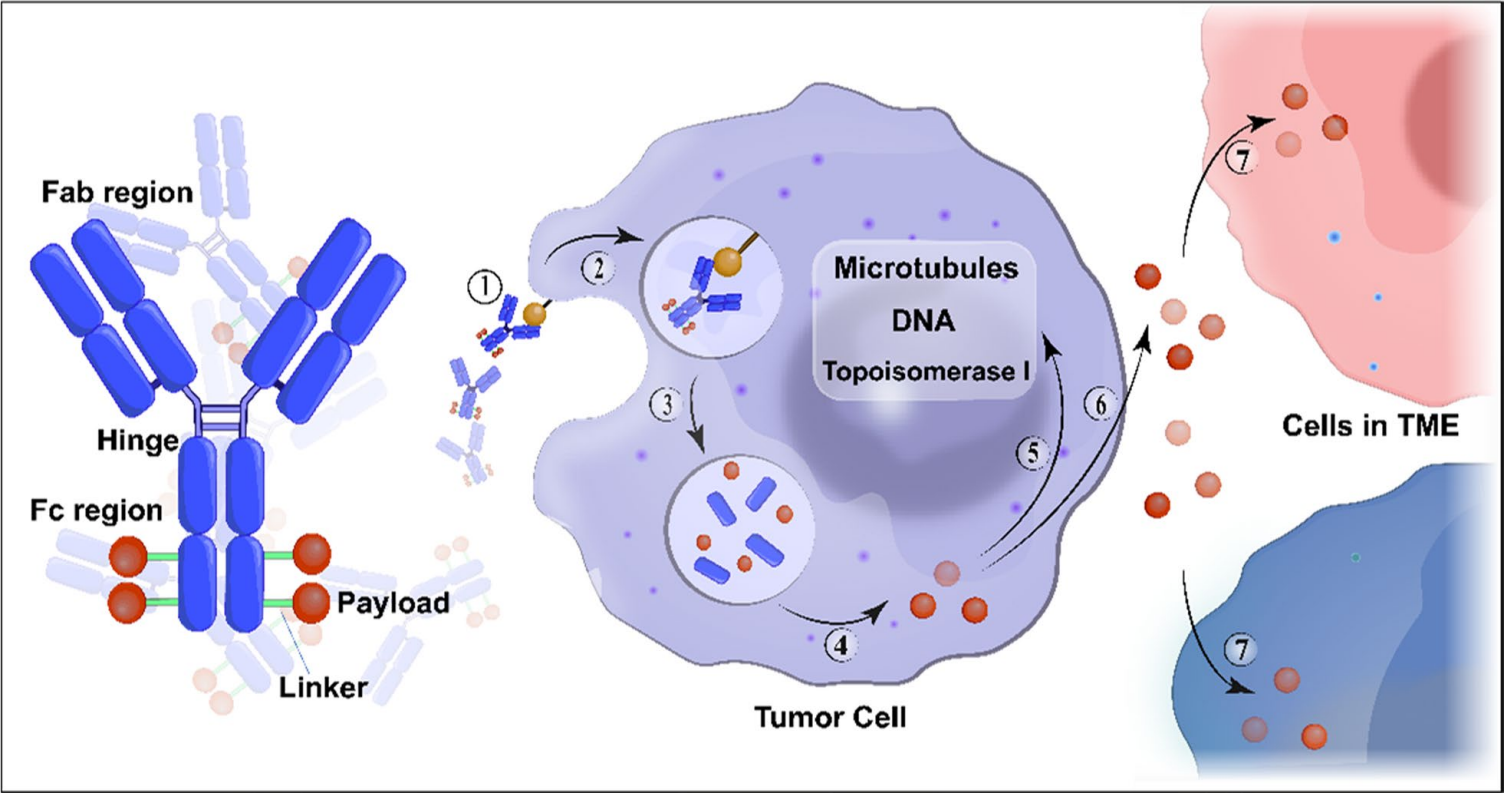
Structure and mechanism of action of conventional ADCs. ADCs consist of three essential components: a monoclonal antibody that binds to an antigen primarily expressed on the surface of tumor cells, providing specificity in targeting tumor cells; a linker that prevents premature release of the payload in the bloodstream but instead releases it in the tumor cells; and a cytotoxic payload that triggers tumor cell death by targeting critical components such as DNA, microtubules, and topoisomerase. ADC cytotoxicity involves a series of sequential stages: ① binding of the antibody to the antigen, ② internalization of the ADC-antigen complex, ③ degradation of the ADC in the lysosomes, ④ release of the payload in the cytoplasm, ⑤ its interaction with the target; ⑥ possible discharge of a fraction of the payload into the extracellular milieu, ⑦ subsequent occurrence of the bystander effect where it is internalized by neighboring cells in the tumor microenvironment. Abbreviation: TME, tumor microenvironment

In the past decade, antibody-drug conjugates (ADCs) have emerged as a transformative treatment modality for a broad spectrum of solid and hematological malignancies [1, 2]. ADCs are antibody-based macromolecular complexes comprising three main constituents: antibodies, linkers, and payloads. Their mechanism of action can be summarized as follows: when the antibody binds to the antigen on the surface of a target cell, the ADC is internalized, releasing the payload and exerting cytotoxicity [3] (Fig. 1). Following the initial approval of ADCs for solid tumors in 2013 [4], interest in this field has increased, and numerous such conjugates have been evaluated across various tumor categories.

Several ADCs have shown potent anti-tumor activities against treatment-refractory cancers. To date, eight ADCs have been approved for solid tumors with different indications (Table 1). Nevertheless, even for target-positive tumor types, most patients do not achieve long-lasting disease control and develop resistance to ADCs. Thus, for many tumor types, a single treatment is insufficient and many ADCs are undergoing clinical trials with more responsive regimens.

**Table 1:** Primary specifics of ADCs currently approved worldwide in treating solid tumors

Drug name	Target	mAb	Linker	Payload	Indications	Year of Approval	Efficacy	DAR	Regimen
Kadcyla® (ado-trastuzumab emtansine)	HER2	IgG1	SMCC	DM1	HER2 + metastatic BC previously treated with trastuzumab & a taxane	2013	T-DM1 vs. capecitabine-lapatinib; mOS 30.9 vs. 25.1 mo (HR 0.65; 95% CI 0.64–0.88); mPFS 9.6 vs. 6.4 mo (HR 0.65; 95% CI 0.55–0.77)	3.5	3.6 mg/kg, Q3W
					HER2 + early BC after neoadjuvant taxane & trastuzumab-based treatment	2019	T-DM1 vs. trastuzumab; 3y iDFS 88.3% vs. 77% (HR 0.5; 95% CI 0.39–0.64)		
Enhertu®(famtrastuzumab deruxtecannxki)	HER2	IgG1	GGFG	DXd	Unresectable or metastatic HER2 + BC after 2 or more anti-HER2 regimens	2019	Single-arm ORR = 61.4%	8	6.4 mg/kg, Q3W
					Locally advanced or metastatic HER2 + gastric or gastroesophageal junction adenocarcinoma after a trastuzumab-based regimen	2021	T-DXd vs. CT, ORR: 51% vs. 14%		
Aidixi ®(Disitamab vedotin)	HER2	IgG1	MC-VA-PABC	MMAE	HER2 + (IHC 3 + or 2 +) locally advanced or metastatic UC who previously failed at least one line of CT	2020	Single-arm ORR = 51.2%; mPFS = 6.9mo	4	2.5 mg/kg, Q2W
					HER2-overexpressing (IHC 2 + or 3 +), locally advanced or metastatic GC/GEJC who were under at least second-line therapy	2021	Single-arm ORR = 24.8%		
Padcev® (enfortumab vedotin-ejfv)	Nectin-4	IgG1	MC-VC-PABC	MMAE	Locally advanced or metastatic UC who were previously treated with platinum CT and anti-PD-1/L1 therapy	2019	Single-arm ORR = 44%	3.8	1.25 mg/kg on days 1, 8 and 15, Q4W
Trodelvy® (sacituzumab govitecan hziy)	TROP2	IgG1	CL2A	SN38	Locally advanced or metastatic TNBC after at least two prior therapies	2020	SG vs.CT; mPFS 5.6 vs. 1.7 mo (HR 0.41; 95% CI 0.32–0.52)	7.6	10 mg/kg, days 1 and 8, Q3W
Akalux® (cetuximab saratolacan sodium)	EGFR	IgG1	N/A	IRDye/700DX	Unresectable locally advanced or recurrent head and neck cancer	2020	Single-arm ORR = 26.7%	NA	640 mg/m^2^, and the light dose was fixed at 50 J/cm^2^ for superficial tumors and 100 J/cm^2^ fiber diffuser length for interstitial tumors
Tivdak®(tisotumab vedotin-tftv)	TF	IgG1	MC-VC-PABC	MMAE	Recurrent or metastatic cervical cancer with disease progression on or after CT	2021	Single-arm ORR = 24%	4	2 mg/kg (up to a maximum of 200 mg for patients weighing ≥ 100 kg), Q3W
Elahere®(mirvetuximab soravtansine-gynx)	FRα	IgG1	sulfoSPDB	DM4	High-grade serous epithelial ovarian cancer, primary peritoneal, or fallopian tube cancer	2022	Single-arm ORR = 32.4%	3.4	6 mg/kg using adjusted ideal body weight, Q3W

T-DM1, Ado-trastuzumab emtansine; T-DXd, Fam-trastuzumab deruxtecan; SG, sacituzumab govitecan; IgG, immunoglobulin G; PD-1/L1, programmed cell-death protein 1 (PD-1)/programmed cell-death ligand 1 (PD-L1); HER2, human epidermal growth factor receptor 2; TROP2, trophoblast cell surface antigen 2; DM1, maytansinoid antitubulin; DXd, deruxtecan, camptothecin, topoisomerase-I inhibitor; TF, tissue factor; DAR, drug-to-Antibody Ratio; MMAE, monomethyl auristatin E; BC, breast cancer; TNBC, triple negative breast cancer; UC, urothelial cancer; CT, chemotherapy; GC/GEJC, gastric/gastro-oesophageal junction cancer; ORR, overall response rate; mOS, median overall survival; mPFS, median progression-free survival; HR, hazard ratio; mo, months; CI, confidence interval; iDFS, invasive disease-free survival; FRα, folate receptor alpha; QnW, every *n* week(s)

In the realm of cancer treatment, it is widely acknowledged that the likelihood of achieving complete remission and cure is often heightened by combining therapeutic agents that operate through diverse mechanisms of action, particularly when dealing with the complexities of tumor heterogeneity [5]. The primary approach for addressing resistance and/or enhancing ADC therapies involves the integration of ADCs with different therapeutic strategies. Synergy is commonly defined as the effect of two or more agents working in combination that is greater than the expected additive effect. An additive effect is generally considered as the baseline effect for synergy detection methods. Consequently, active research is exploring the combination of ADCs with various other types of anticancer medications, such as chemotherapy, radiotherapy, endocrine therapy, targeted molecular agents, and immunotherapy, both in preclinical models and clinical trials. There is an interest in developing rational combinations that could prolong survival compared to monotherapies.

In this review, we discuss the mechanisms of different ADC combination therapies and review the ongoing clinical trials for their selection and evaluation. Finally, we outline and examine key translational, statistical, and regulatory considerations from a combination perspective, highlighting the current progress and significant challenges yet to be addressed.

## ADCs combined with chemotherapy

Integrating different forms of chemotherapy with ADC has proven to be a well-accepted strategy for overcoming drug resistance and achieving favorable treatment outcomes in preclinical and clinical studies [6]. Exploring the most effective combination regimen requires a comprehensive understanding of how ADC antibodies and payloads work synergistically with chemotherapy drugs to affect the cell cycle and alter the presence of surface antigens. However, to date, many ADCs have been added to commonly used chemotherapeutic regimens merely as carriers for the delivery of toxic payloads without considering their synergistic effects, leading to mixed results in both preclinical and clinical research. This highlights the significant and unmet need for continued efforts in designing clinical trials for ADCs combined with chemotherapy. Table 2 presents a list of such trials.

**Table 2:** Summary of clinical trials investigating the combination of ADCs and chemotherapy

Target	NCT number	Other names	Drug	Partner drugs	Partner drug category (target)	Phase	Start	Treatment setting	Efficacy
HER2	NCT01702558 [19]	TRAXHER2	T-DM1	Capecitabine	CT	I	2012	mBC, mGC	Negative
	NCT02073916 [20]	STELA	T-DM1	Lapatinib + Abraxane	EGFR/HER2 TKI + CT	I	2013	mBC	Positive
	NCT02073487 [21]	TEAL	T-DM1	Lapatinib + Abraxane	EGFR/HER2 TKI + CT	II	2014	Neoadjuvant, BC	Positive
	NCT02562378 [22]	THELMA	T-DM1	Non-pegylated Liposomal Doxorubicin	CT	I	2015	mBC	Negative
	NCT03190967 [23]	NA	T-DM1	TMZ	CT	I/II	2017	mBC	Terminated
	NCT04686305 [24]	DL03	T-DXd	Durvalumab and Cisplatin	IO + CT	Ib	2020	mNSCLC	NA
				Durvalumab and Carboplatin	IO + CT				NA
				Durvalumab and Pemetrexed	IO + CT				NA
				Durvalumab and Cisplatin	IO + CT				NA
				Durvalumab and Carboplatin	IO + CT				NA
				Durvalumab and Pemetrexed	IO + CT				NA
				Durvalumab	IO				NA
TROP2	NCT05687266 (recruiting)	AVANZAR	Datopotamab deruxtecan	Durvalumab + Carboplatin	IO + CT	III	2022	mNSCLC	NA
Nectin-4	NCT03288545 [25]	EV-103	Enfortumab vedotin	Pembrolizumab	IO	I/II	2017	mUC	Positive
				Cisplatin	CT				NA
				Carboplatin	CT				NA
				Gemcitabine	CT				NA
				Platinum + Pembrolizumab	IO + CT				NA
				Pembrolizumab	IO			MIBC	NA
TF	NCT03485209 [26]	InnovaTV 207	Tisotumab Vedotin	Pembrolizumab + (Carboplatin or DDP)	IO + CT	II	2018	Advanced solid tumors	NA
				Pembrolizumab	IO				NA
EGFR	NCT02573324 [27]	Intellance1	Depatuxizumab Mafodotin	TMZ and Radiation	CT + Radiation	III	2015	GBM	Positive
NaPi2b	NCT04907968 (active, not recruiting)	UPGRADE	Upifitamab Rilsodotin	Carboplatin	CT	I	2021	High grade serous ovarian cancer	Terminated
FRα	NCT02606305 [28]	NA	Mirvetuximab Soravtansine	Bevacizumab	Anti-VEGF mAb	Ib/II	2022	High-grade epithelial ovarian, primary peritoneal, or fallopian tube cancers	Positive
				Carboplatin	CT				NA
				Pegylated Liposomal Doxorubicin	CT				NA
				Pembrolizumab	IO				NA
				Bevacizumab + Carboplatin	CT + Anti-VEGF mAb				NA

T-DM1, Ado-trastuzumab emtansine; T-DXd, fam-trastuzumab deruxtecan; HER2, human epidermal growth factor receptor 2; TROP2, trophoblast cell surface antigen 2; TF, tissue factor; mBC, metastatic breast cancer; mTNBC, metastatic triple negative breast cancer; mUC, metastatic urothelial cancer; CT, chemotherapy; IO, Immunotherapy; TKI, Tyrosine kinase inhibitor; mGC, metastatic gastric cancer; TMZ, temozolomide; 5-FU,5-fluorouracil; FRα, folate receptor alpha; GBM, glioblastoma; MIBC, muscle invasive bladder cancer; EGFR, epidermal growth factor receptor; mNSCLC, metastatic non-small cell lung cancer; mAb, monoclonal antibodies; DDP, cisplatin; NA, not applicable

### Mechanism of ADCs combined with chemotherapy

According to reported findings, chemotherapy and ADCs act synergistically in ways that include targeting different phases of the cell cycle or modulating tumor cell surface antigen expression.

### Cell cycle phase blockers

Many chemotherapeutic drugs are DNA-damaging agents, such as antimetabolites, platinum-based compounds, and topoisomerase inhibitors that target the S phase of the cell cycle and induce G2/M arrest, which can be effectively combined with ADC containing microtubule-disrupting payloads that target the G2/M phase of the cell cycle. This concept has been illustrated through the effective combination of carboplatin with mirvetuximab soravtansine (targeting folate receptor α with DM4), anetumab ravtansine (targeting mesothelin with DM4), or luveltamab tazevibulin (targeting folate receptor α with SC239) in ovarian cancer preclinically [7–9]. During early phase trials investigating the synergistic effects of ravtansine-based ADCs in combination with carboplatin or doxorubicin, positive treatment responses were observed in both platinum-sensitive and -resistant patients with ovarian cancer [10–14].

### Improved surface-antigen expression

The choice of chemotherapeutic companion may affect the levels of surface antigens targeted by ADCs. For instance, gemcitabine can upregulate HER2 expression on pancreatic adenocarcinoma cells by 14.81 folds, predominantly within the G2/M phase. Thus, the effect of gemcitabine on DNA synthesis renders it effective against G1 and early S phase cells, whereas G2/M phase cells are more resistant. The enhanced HER2 expression in G2/M cells implies a greater likelihood of gemcitabine effectively binding with trastuzumab emtansine (T-DM1, HER2 targeted with DM1 payload), which contributes to the improved efficacy of the combination on pancreatic ductal adenocarcinoma cells [15]. Thus, gemcitabine generate synergistic effects in combination with T-DM1 through their ability to enhance antigen availability. However, it remains uncertain whether this observation holds true for other ADC-chemotherapy combinations with different targets, and whether the increased antigen expression levels are directly related to the actual available antigenic epitopes for ADC binding or even to the efficacy of ADCs.

### Coordination of different drugs

The timing of administration is a significant factor to consider when designing ADC combinations, as most conjugates must be internalized by tumor cells to be effective, which involves systemic transport and cell entry processes. For example, induction of G2/M phase arrest by DNA damage requires at least 15 h for microtubule disruptors to act [16]. Wahl et al. elegantly demonstrated this concept in preclinical models of colon, lung, and breast cancers. They observed that sequential management of SGN-15 (a construct targeting the Lewis Y antigen with a doxorubicin payload) followed by paclitaxel resulted in greater DNA fragmentation than simultaneous treatment [17].This observation suggests that the sequence of drug administration may be taken into consideration when combined chemotherapy with ADCs. However, these concepts await clinical trial assessment and should be explored in light of the recognized rates of ADC internalization and cell cycle progression in individual tumor types.

### Safety profile of the ADC–chemotherapy combination

Notably, the combination of ADCs and chemotherapy presents challenges related to overlapping toxicities. Substantial insights in this regard have been gained from clinical trials. For example, a study evaluating T-DM1 in combination with docetaxel (with or without pertuzumab) for HER2-positive breast cancer demonstrated dose-limiting toxicities (DLTs) and grade ≥ 3 adverse events in approximately 80% patients with metastatic breast cancer [18]. These adverse effects included neutropenia, fatigue, epistaxis, stomatitis, nausea, and diarrhea. Similarly, the combination of T-DM1 with capecitabine resulted in increased discontinuation rates without a significant improvement in response rates [19]. The combination of trastuzumab deruxtecan (T-DXd) with 5-fluorouracil (5-FU) or capecitabine resulted in notable toxicities in patients with metastatic HER2-positive gastric cancer, with dose-limiting stomatitis and a high incidence of grade ≥ 3 adverse events in DESTINYGastric03 trial [14].

Datopotamab deruxtecan (Dato-DXd) in combination with platinum-based chemotherapy and pembrolizumab resulted in substantial grade ≥ 3 toxicities in a significant proportion of patients, characterized by the common occurrence of nausea, anemia, fatigue, and stomatitis [13]. In addition, the combination of mirvetuximab soravtansine with carboplatin in a Phase Ib trial resulted in notable toxicities including nausea, vomiting, diarrhea, eye problems, fatigue, and cytopenia [10].

In summary, the results of these studies suggested a notable increase in toxicity when ADCs were combined with conventional chemotherapy. This is likely due to the overlap of toxicities resulting from the off-target and off-tumor effects of the ADC payloads.

## ADCs combined with endocrine therapy

Endocrine therapy is a widely used therapeutic approach for hormone-sensitive cancers (e.g., breast and prostate cancers). It works either by blocking hormone synthesis or interfering with hormones that stimulate the growth of tumor cells. Both ADCs and endocrine therapy drugs can induce cellular effects that jointly impede tumor cell survival and proliferation. Combination therapy reduces the likelihood of tumor cells developing resistance by employing multiple drugs with distinct mechanisms of action. There have been some clinical trials related to ADC combined with endocrine therapy (Table 3), while basic research to explore the mechanism of combined action is lacking.

**Table 3:** Summary of clinical trials investigating the combination of ADCs and endocrine therapy

Target	NCT number	Other names	Drug	Partner drugs	Partner drug category	Phase	Start	Treatment setting	Efficacy
HER2	NCT01772472 [30]	KATHERINE	T-DM1	Unspecified	ET	III	2013	Adjuvant, BC	Positive
	NCT04556773 (active, not recruiting)	DB-08	T-DXd	Anastrozole or Fulvestrant	ET	Ib	2020	mBC	NA
	NCT04553770 [31]	NA	T-DXd	Anastrozole	ET	II	2020	Neoadjuvant, BC	NA
HER3	NCT05569811 (active, not recruiting)	VALENTINE	Patritumab deruxtecan	Letrozole	ET	II	2022	Neoadjuvant, BC	NA

T-DM1, Ado-trastuzumab emtansine; T-DXd, fam-trastuzumab deruxtecan; HER2, human epidermal growth factor receptor 2; HER3, human epidermal growth factor receptor 3; mBC, metastatic breast cancer; ET, endocrine therapy; NA, not applicable

### Safety profile of the ADC–endocrine therapy combination

The side effects of endocrine therapy are minimal [29]. This gives clinicians more confidence in adding ADC drugs to endocrine therapy, as demonstrated in the KATHERINE phase III clinical trial. In this trial, the adjuvant utilization of T-DM1 was compared with that of trastuzumab in patients with HER2-positive breast cancer with residual disease who had undergone neoadjuvant HER2-targeted therapy. Both treatment arms were permitted to include concurrent adjuvant endocrine therapy. Patients administered T-DM1 with or without endocrine therapy exhibited comparable toxicity rates of any grade. Similarly, no significant differences between the two groups were observed in terms of grade ≥ 3 adverse events (26.0% versus 24.9%), serious adverse events (12.9% versus 12.2%), and events that resulted in a T-DM1 dosage reduction (11.0% versus 15.0%) [30].

The possibility of combining endocrine therapy with T-DXd has been investigated in patients with HER2-low breast cancer at both early and advanced stages. The TALENT study is a randomized phase II trial evaluating the administration of neoadjuvant T-DXd, with or without anastrozole, in patients with early breast cancer and low HER2 expression. Interestingly, this study showed that both treatment arms exhibited similar toxicity profiles, highlighting the feasibility of this combination approach [31]. Similarly, the Phase Ib study, DESTINY-Breast08, revealed that adding anastrozole or fulvestrant to T-DXd did not result in any DLTs. This combination approach was observed to maintain a toxicity profile akin to that of the solitary administration of T-DXd in individuals diagnosed with metastatic HER2 low breast cancer.

In general, the co-administration of ADCs and endocrine therapy does not appear to result in increased toxicity. This observation is consistent with the distinct patterns of adverse effects exhibited by each agent when administered independently. This is also consistent with the favorable safety profiles of most endocrine therapies compared to other systemic cancer treatments.

## ADCs combined with radiotherapy

The combination of radiotherapy and ADC includes external radiation therapy combined with ADC and radionuclide antibody conjugates (RACs). RACs, also known as radioimmunoconjugates, radioimmunotherapy, or targeted radiotherapy, are a type of medical treatment that uses specific monoclonal antibodies labelled with radioactive isotopes (radionuclides, generally beta emitters), as described in a review by Mattes [32]. Therefore, RAC is not discussed in the present review. Based on the timing of radiotherapy and ADC administration, the combination is either concomitant or sequential. Concomitant radiotherapy involves simultaneous administration of ADC and radiotherapy. However, the definition of sequential ADC administration varies across studies: the temporal span ranges from 77 to 131 days when ADC is administered before radiotherapy and 420 to 1426 days when administered after radiotherapy [33]. The fractionation regimens include conventional fractionated radiotherapy and stereotactic radiosurgery (SRS)/stereotactic body radiotherapy (SBRT). The clinical studies on ADCs combined with radiotherapy are shown in Table 4.

**Table 4:** Summary of clinical trials investigating the combination of ADCs and radiotherapy

NCT number	Start	Type of study	No	RT site	RT type	RT prescription	Drug	Timing	Treatment setting	Toxicity (Radiation-related)	Efficacy
NCT01196052 [34]	2010	Phase II (subgroup)	116	Beast/chest wall/regional node	CFRT	RT prescription according to local standards	T-DM1 ± Trastuzumab	Con/Seq (pre/post-RT)	HER2 + early-stage BC received (neo)adjuvant	NR	NA
NCT01772472 [35]	2013	Phase III (subgroup)	624	Beast/chest wall/regional node	CFRT	RT prescription according to local standards	T-DM1	Con	HER2 + early BC patients undergoing breast-conserving surgery or that with T3N + or T4 and/or with N2-3	RT-related skin injury, 13.2% grade 1; 10.8% grade 2; 1.4% grade 3 or more. Radiation pneumonitis, 0.41% grade 1; 0.82%grade 2; 0.27% grade3. Pulmonary radiation injury, 0.14% grade 1	Positive
Julie A. Carlson [36]	2014	Case series	7	Tumor	SRS	16–24 Gy/1F	T-DM1	Seq (3 d-49 d post-RT)	HER2 + BC patients with brain metastases	Radiation necrosis 57%	NA
NCT02573324 [37]	2015	Phase III	639	Tumor/TB	CFRT	60 Gy/30 F or 59.4 Gy/33 F	TMZ + Depatux-m	Con	EGFR-amplification newly diagnosed GBM	Seizure, 20.4% any grade; 5% grade 3; 0.6% grade 4. No specific radiation-related toxicity was reported	Negative
NCT02590263 [38]	2015	Phase I/II	15	Tumor/TB	CFRT	60 Gy/30 F in 42 days	TMZ + Depatux-m	Con	EGFR-amplification newly diagnosed WHO grade III/IV glioma	RT-related skin injury, 73% ≥ grade3. Headache, 20% ≥ grade3	
											NA
A Géraud [39]	2016	Case series	3	Dorsal vertebras; sacrum; left shoulder	SRS	15 Gy/5 F or 8 Gy/1 F	T-DM1	Con	HER2 + metastatic BC	No side effects	Positive
William Jacot [40]	2016	Retrospective study	36	Tumor/whole brain	SRS/WBRT	NR	T-DM1	Seq (pre-RT)	HER2 + BC patients with brain metastases	NR	Positive
Arthur Geraud [41]	2017	Retrospective study	12	Tumor ± whole brain	SRS ± WBRT	NR	T-DM1	Con/Seq (pre-RT)	HER2 + BC patients with brain metastases	Radiation necrosis, 50% in Con group and 28.6% in Seq group. Other toxicity, alopecia; memory disorders; locomotor disorders; balance disorders; and visual disorders	NA
Zita Zolcsák [42]	2020	Retrospective study	14	TB/Breast/ chest wall	CFRT	50 Gy/25 F ± boost of 14.5 Gy-16 Gy to TB	T-DM1	Con	Nonmetastatic HER + BC patients with residual invasive disease following neoadjuvant therapy	Radiodermatitis, 86% grade 1; 14% grade 2; RT-related fatigue, 14% grade 1; Pain in the irradiation zone, 7% grade 1; LVEF decrease, 14% grade 2	NA
Matthew N. Mills [43]	2021	Retrospective study	16	Tumor	SRS	21 Gy (14 Gy-24 Gy)/1 F or 25 Gy (20 Gy-30 Gy)/13-5 F	T-DM1	Con/Seq (pre/post-RT)	HER2 + BC patients with brain metastases	Radiation necrosis, 3%. Other toxicity, headaches and fatigue	Positive
NCT05979740 (recruiting)	2023	Phase II	6	Bladder	CFRT	≥ 50 Gy in about 30 F	RC48 + PD-1	Con	MIBRC with high HER2 expression (IHC 2 + or 3 +) and could be treated with bladder conserving therapy with maximum TURBT	NR	NA

RT, radiotherapy; CFRT, conventional fractionated radiotherapy; WBRT, whole brain radiotherapy, SRS, stereotactic radiosurgery; TB, tumor bed; Con, concurrent; Seq, sequential; NR, not reported; LN, lymph node; LVEF, left ventricular ejection fraction; Depatux-m, depatuxizumab mafodotin; TMZ, temozolomide; F, fractions; BC, breast cancer; No., sample size; TURBT, transurethral resection of bladder tumor, GBM, glioblastoma; MIBRC, muscle invasive bladder uroepithelial cancer; WHO, World health orgnization; NA, not applicable

### Mechanism of ADCs combined with radiotherapy

The synergistic mechanisms of the combined application of radiotherapy and ADC include the regulation of surface antigen expression in tumor cells by radiotherapy, an increase in radiation sensitivity of tumor cells by ADC, and other potential mechanisms, such as affecting the tumor microenvironment (TME) and vascular permeability.

### Radiation induces generation of (neo)antigens

Ionizing radiation (IR) induces morphological and functional alterations in tissues [44]. Tumor cells are more prone to survival and propagation when “stress-regulated proteins” are highly upregulated by external stimuli. (Neo-) antigens expressed on the surface of cancer cells after IR exposure provide opportunities to develop cancer-targeted therapeutics. Cell adhesion molecules were the first IR-inducible proteins identified [45, 46]. However, these inducible proteins are expressed on microvascular endothelial cells rather than on tumor cells, and some are shed from the cell surface. Glucose-regulated protein 78 (GRP78) is a radiation-induced endoplasmic reticulum stress response protein that plays an important role in radioresistance, enhancement of tumor cell proliferation, protection against apoptosis, and promotion of tumor angiogenesis [47, 48]. The effectiveness of combining GRP78 antibodies with radiotherapy has been studied previously [49, 50]. The researchers discovered that antibodies targeting the functional domain of GRP78 disrupt its interactions with its binding partners. Consequently, this reduces tumor cell viability and enhances radiosensitization. Tax interacting protein 1 (TIP-1), another radiation-induced tumor-specific target, translocates to the cell plasma membrane after exposure to IR [51]. Based on this finding, Lewis et al. conjugated a high-affinity anti-TIP-1 antibody (7H5) to a payload such as monomethyl auristatin E (MMAE) with a valine-citrulline (Vc) linker to form a radiosensitizer (7H5-VcMMAE) [52]. The use of 7H5-Vc-MMAE in combination with radiotherapy resulted in a prolonged delay in tumor growth and improved the survival of A549 and H1299 non-small cell lung cancer (NSCLC) animal models. To date, the targeting of radiation-inducible antigens using ADCs combined with radiotherapy has not been explored in clinical trials. The new clinical paradigm of using IR to guide drug delivery merits further investigation in preclinical and clinical trials involving patients with radiation-resistant cancers.

### ADCs increase the sensitivity of tumor cells to radiotherapy

The cell cycle phase plays an important role in determining the relative radiosensitivity of cells [53]. Cells are most sensitive to IR during the G2/M phase, display intermediate sensitivity in the G1 phase, and exhibit the lowest sensitivity in the later stages of the S phase. Based on these findings, many radiosensitizing drugs have been developed to increase the anti-tumor activity and optimize patient outcomes. However, in practice, the clinical utility of radiosensitizing drugs is substantially curtailed due to unintended off-target side effects. To address this critical challenge, radiation-sensitizing payloads (e.g., MMAE, MMAF, and DM1) have been conjugated to antibodies to selectively radiosensitize tumors based on antigen overexpression. These radiosensitizer-ADCs are capable of increasing radiosensitizer delivery to tumors, enhancing radiation-induced cytotoxicity, and improving tumor control [54, 55]. Furthermore, in combination with radiotherapy, radiosensitizer-ADCs have been shown to enhance the effectiveness of radiation and improve survival in preclinical tumor models of the lung, head and neck, oesophageal, breast, and pancreatic cancers [52, 54–58].

### Other potential mechanisms

Radiotherapy not only directly acts on tumor cells but also affects the TME in a complex and dynamic manner. Several radiation-induced molecules within tumor blood vessels, including ICAM-1, E-selectin, P-selectin, and β3 integrin, reportedly have the potential to serve as therapeutic targets [59, 60]. Moreover, there is mounting evidence that the interplay between radiotherapy and the TME could be utilized to enhance the accumulation and intratumoral distribution of nanoparticles and liposome formulations mediated by changes in the vasculature and stroma, with secondary effects on hypoxia, interstitial fluid pressure, solid tissue pressure, and the recruitment and activation of bone marrow-derived myeloid cells [61–63]. A previous study found that vascular permeability in tumors significantly increased 24 h after irradiation at doses higher than 400 cGy, resulting in increased antibody uptake following radiation [64]. In addition to affecting the tumor blood vessels, radiotherapy also has a direct impact on blood–brain barrier permeability. Nakata et al. found that a single large dose of 20–40 Gy promoted the extravasation of serum albumin in the rat brain tissue preclinically [65]. Nevertheless, whether conventional fractionated radiotherapy, routinely used in clinics, can promote ADC penetration of the blood–brain barrier remains unknown and the underlying mechanism remains elusive. Finally, the effect of radiotherapy on the distribution of antibodies and the payload of linker-cleavable ADCs in tumors is yet to be investigated.

In summary, multiple potential synergistic benefits and underlying mechanisms of the combination of radiotherapy and ADCs deserve further exploration at both the preclinical and clinical stages.

### Safety profile of the ADC–radiotherapy combination

The clinical applications of radiosensitizer-ADCs have also been evaluated. For non-CNS tumors, there is insufficient robust evidence regarding the safety profile and efficacy of ADCs in combination with different radiotherapy segmentations. Available data mainly focus on breast cancer treatments [33, 66]. The KATHERINE trial evaluated the effectiveness of adjuvant T-DM1 in patients with HER2-positive breast cancer and residual disease after neoadjuvant chemotherapy with anti-HER2 therapy [35]. The results demonstrated that adjuvant T-DM1 treatment reduces the risk of disease recurrence and death (50%). In this clinical trial, patients who underwent breast-conserving surgery and those who had locally advanced disease following mastectomy (clinical T_3_N_+_/T_4_N_x_ /T_x_N_2-3_ disease) were administered radiation within 60 days of surgery. However, a subgroup analysis specific to patients undergoing radiation was not conducted to assess the safety profile of the combined treatment. An increase in ≥ 3 grade toxicities was noted among the irradiation group in comparison to the non-irradiated group (27.4% vs. 16.2%). Comparable incidences of radiation-related cutaneous complications were observed, affecting 25.4% and 27.6% patients in the T-DM1 and trastuzumab groups, respectively. Patients who were administered T-DM1 showed a modest increase in the occurrence of radiation-induced pneumonitis and pulmonary radiation injury rates (1.5% and 0.1%, respectively), in contrast to those administered trastuzumab (0.7% and 0%) [30]. Considering the potential risk of cardiotoxicity associated with trastuzumab, a study was conducted to investigate the cardiac safety and feasibility of radiotherapy in combination with T-DM1 in a group of 116 patients (*n*_concurrent_ = 39, *n*_sequential_ = 77) [34]. Roughly 95% patients receiving T-DM1 plus radiotherapy successfully adhered to ≥ 95% of the planned radiotherapy dosage with a delay of ≤ 5 days. No protocol-prespecified cardiac side effects or instances of heart failure were reported following T-DM1. However, Zolcsak et al. presented the initial safety profile associated with the concurrent use of T-DM1 and radiotherapy in a group of 14 patients diagnosed with residual invasive HER2-positive breast cancer. A dosage of 50 Gy delivered in 25 fractions was administered for adjuvant irradiation of the breast or chest wall. A reversible grade 2 decrease in left ventricular ejection fraction (LVEF) was observed in two patients [42]. Considering the mechanism of T-DM1’s action involving radiosensitization through microtubule inhibitors and in the absence of solid safety data, the delivery of concurrent radiation should be approached with caution. Recent clinical studies have revealed that T-DXd improved both progression-free survival (PFS) and overall survival (OS) compared to T-DM1, but information concerning the combined use of T-DXd and radiotherapy is scarce. T-DXd exhibited an increased incidence of drug-related interstitial lung disease (ILD) or pneumonitis (10.5% vs. 1.9%), and gastrointestinal toxicities were more frequently reported with T-DXd treatment. The combination of T-Dxd with thoracic or abdominal radiotherapy requires extreme caution. In a metastatic breast cancer setting, a case-series study assessed the toxicity of concurrent palliative radiotherapy and T-DM1 in three patients with bone metastases [39]. The radiotherapy field involved the thoracic vertebrae, sacrum, and shoulder, with a prescribed dose of 15 Gy delivered in five fractions or 8 Gy delivered in one fraction. All patients experienced substantial pain relief, and no documented adverse reactions associated with the concomitant use of radiotherapy and T-DM1 were reported. Furthermore, approximately 50% of HER2-positive metastatic breast cancer cases are associated with brain metastases [67]. In terms of CNS metastatic tumors, high-level data on the effectiveness and tolerance of concurrent administration of ADCs and brain radiation therapy are insufficient. Evidence gleaned from case reports or a small series of patients indicates that the combination of T-DM1 and concomitant whole-brain radiation therapy is manageable, without severe side effects or any increase in clinically significant toxicity [40]. However, prudence is needed when considering concurrent or sequential SRS as several cases of complications have been documented in the literature [36, 41, 43, 68, 69]. The case series presented by Carlson et al. showed that four out of seven (57.1%) patients who underwent SRS after T-DM1 administration developed radiation brain necrosis [36]. This elevated rate of clinical radiation brain necrosis is clearly unacceptable. In another study involving 45 patients diagnosed with CNS metastases from breast cancer, Stumpf et al. observed a 13.5-fold rise in the risk of radiation necrosis when T-DM1 was administered in conjunction with SRS [68]. The DEBBRAH phase II study demonstrated the feasibility of combining intracranial treatment with T-Dxd and radiation, showing manageable toxicity in patients with HER2-positive and HER2-low breast cancer who underwent whole-brain radiation therapy and/or SRS. However, the authors did not mention the timing between irradiation and the sequential administration of T-DXd [70]. Notably, significant heterogeneity was observed among these studies. Regarding primary CNS tumors, there are already some data on the effectiveness and safety outcomes of the combination of ADC and radiation treatment. An anti-EGFR antibody conjugated to MMAF, Depatuxizumab-mafodotin (Depatux-M), was administered to patients with glioblastoma (GBM) receiving standard treatment with radiotherapy plus temozolomide [37]. The safety profile of Depatux-M combined with radiotherapy and temozolomide for the treatment of newly diagnosed GBM is acceptable. However, interim analysis revealed that Depatux-M did not yield an OS benefit for the treatment of newly diagnosed EGFR-amplification GBM, notwithstanding the longer PFS. The study was terminated at the early stage. The potential reasons for these negative results are as follows: 1. Depatux-M may be ineffective in treating GBM; 2. there is a probability that Depatux-M effectively eliminated EGFR-amplification (and particularly EGFRvIII-mutant) tumor cells, improving PFS; however, resistant clones emerged and voided any OS benefit, a hypothesis supported by results from patient-derived xenografts [71]; 3. heterogeneous delivery across the blood–brain barrier limits the efficacy of Depatux-M in CNS tumors [71], and non-cleavable linkers are detrimental to drug diffusion within the tumors.

In summary, radiosensitizer-ADCs combined with radiation are a promising treatment strategy; however, there is an urgent need for high-level evidence regarding their safety.

## ADCs combined with molecular targeted cancer therapy

Targeted therapies, including monoclonal antibodies, tyrosine kinase inhibitors (TKIs), and anti-angiogenic agents, have been used in clinical practice for decades to treat tumors with specific mutations, overexpression, and amplification, with clinically proven safety and efficacy. However, the efficacy of these treatments in combination with ADC remains poorly understood. In this section, we focus on the treatment strategies that combine ADCs with targeted therapeutics and discuss their potential synergistic effects and safety profiles. The corresponding clinical trials are shown in Table 5.

**Table 5:** Summary of clinical trials investigating the combination of ADCs and targeted therapy

Target	NCT number		Other names	Drug	Partner drugs	Partner drug category (target)	Phase	Start	Treatment setting	Efficacy
HER2	NCT01120184 [72]		MARIANNE	T-DM1	Pertuzumab	Anti-HER2 mAb	III	2010	mBC	Positive
					Taxane	CT				
	NCT03225937 [73]		HERACLES	T-DM1	Pertuzumab	Anti-HER2 mAb	II	2012	mCRC	Negative
	NCT02073916 [20]		STELA	T-DM1	Lapatinib + Abraxane	EGFR/HER2 TKI + CT	I	2013	mBC	Positive
	NCT01983501 [74]		NA	T-DM1	Tucatinib	HER2 inhibitor	Ib	2014	mBC	Positive
	NCT02038010 [75]		NA	T-DM1	BYL719 (alpelisib)	PI3Kα inhibitor	I	2014	mBC	Positive
	NCT02073487 [21]		TEAL	T-DM1	Lapatinib + Abraxane	EGFR/HER2 TKI + CT	II	2014	Neoadjuvant, BC	Positive
	NCT02657343 [76]		NA	T-DM1	Ribociclib	CDK4/6 inhibitor	Ib/II	2016	mBC	Negative
	NCT03364348 (completed)		NA	T-DM1	Utomilumab	T cell co-stimulatory receptor agonist (4-1BB)	I	2017	mBC	NA
	NCT03523572 [77]		NA	T-DXd	Nivolumab	IO	I	2018	mBC & mUC	Positive
	NCT04042701 [78]		NA	T-DXd	Pembrolizumab	IO	I	2019	mBC & mNSCLC	NA
	NCT03975647 [79]		HER2CLIMB-02	T-DM1	Tucatinib	HER2 inhibitor	III	2019	mBC	NA
	NCT04264936 [80]		NA	RC48	Toripalimab (JS001)	IO	Ib/II	2020	mUC	Positive
	NCT04235101 (completed)		NA	(Vic-)trastuzumab duocarmazine (SYD985)	Niraparib	PARP inhibitor	I	2020	Advanced solid tumors	NA
	NCT04538742 [81]		Destiny Breast 07	T-DXd	Pertuzumab	HER2 mAb	Ib/II	2020	mBC	NA
					Paclitaxel	CT				
					Durvalumab	IO				
					Tucatinib	HER2 inhibitor				
	NCT04556773 (active, not recruiting)		Destiny Breast 08	T-DXd	Anastrozole	ET	Ib/II	2020	mBC	NA
					Capivasertib	Akt inhibitor			mBC	
					Fulvestrant	ET			mBC	
	NCT04539938 [82]		HER2CLIMB-04	T-DXd	Tucatinib	HER2 inhibitor	II	2020	mBC	NA
	NCT04197687 (recruiting)		NA	T-DM1	TPIV100 + Sargramostim	HER2 vaccine + GM-CSF	II	2020	Adjuvant BC	NA
					Sargramostim	GM-CSF				
	NCT04704661 (recruiting)		DASH	T-DXd	AZD6738	ATR inhibitor	I	2021	Advanced solid tumors	NA
	NCT04983121 (recruiting)		NA	ARX788	Pyrotinib Maleate	Pan-ErbB TKI	II	2021	Neoadjuvant, BC	NA
	NCT04585958 (recruiting)		NA	T-DXd	Olaparib	PARP-1/2 inhibitor	I	2021	mEC	NA
	NCT05372614 (recruiting)		NA	T-DXd	Neratinib	Pan HER inhibitor	I	2022	Advanced solid tumors	NA
	NCT05426486 (recruiting)		NA	ARX788	Pyrotinib	Pan-ErbB TKI	II/III	2022	Neoadjuvant, BC	NA
	NCT05868226 (recruiting)		PRE-I-SPY-PI	T-DXd	ALX148	CD47-blocking fusion protein	I	2022	mBC	NA
TROP2	NCT04039230 [83]		NA	SG	Talazoparib	PARP inhibitor	I/II	2019	mBC	Positive
	NCT04381832 (active, not recruiting)		NA	SG	Etrumadenant + Zimberelimab	Dual adenosine receptor antagonist + IO	I/II	2020	mCRPC	NA
					Etrumadenant	Dual adenosine receptor antagonist				
	NCT05143229 (recruiting)		ASSET	SG	Alpelisib	Specific Pi3K inhibitor	I	2021	mBC	NA
	NCT05006794 (recruiting)		NA	SG	GS9716	MCL-1 antagonist	I	2021	Advanced solid tumors	NA
	NCT05575804 (active, not recruiting)		NA	GQ1001	Pyrotinib	Pan-ErbB TKI	I/II	2022	mBC	NA
Nectin-4	NCT04724018 [84]		NA	EV	SG	ADC	I	2021	mUC	NA
	NCT04878029 (recruiting)		NA	EV	Cabozantinib	MET, RET, AXL, VEGFR2, FLT3, and c-KIT TKI	I	2021	mUC	NA
	NCT03606174 (terminated due to sponsor decision)		NA	EV	Sitravatinib	TYRO3, AXL MER, VEGFR2 and KIT TKI	II	2018	mUC	NA
	NCT04963153 (recruiting) [25]		NA	EV	Erdafitinib	FGFR inhibitor	I	2021	Metastatic bladder cancer	NA
FRα	NCT05200364 [85]		NA	STRO-002	BEV	Anti-VEGF mAb	I	2022	Advanced Epithelial Ovarian Cancer (Including Fallopian Tube or Primary Peritoneal Cancers)	NA
	NCT05445778 (recruiting)		NA	Mirvetuximab soravtansine	BEV	Anti-VEGF mAb	III	2022	Advanced Epithelial Ovarian Cancer (Including Fallopian Tube or Primary Peritoneal Cancers)	NA
			NA		AZD5305	PARP1 inhibitor				
			GLORIOSA		Durvalumab + AZD5305	IO + PARP1 inhibitor				
			NA		5-FU + LV + BEV or Capecitabine + BEV	CT + Anti-VEGF mAb				
MET	NCT02099058 [86]		NA	Telisotuzumab vedotin (ABBV-399)	Osimertinib	EGFR TKI	I/Ib	2014	Advanced solid tumors	NA
			NA		Erlotinib	HER2 mAb				Positive
			NA		Nivolumab	IO				Negative
EGFR-cMET bispecific	NCT05647122 (recruiting)		EGRET	AZD9592	Osimertinib	EGFR TKI	I	2022	Advanced solid tumors	NA
LIV-1	NCT01969643 [87]		NA	Ladiratuzumab vedotin	Trastuzumab	Anti-HER2 mAb	I	2013	mBC	
B7-H3	NCT05293496 (recruiting)		NA	MGC018	Lorigerlimab	Bispecific IgG4 DART	I	2022	Advanced solid tumors	NA

T-DM1, Ado-trastuzumab emtansine; T-DXd, fam-trastuzumab deruxtecan; HER2, human epidermal growth factor receptor 2; HER3, human epidermal growth factor receptor 3; ET, endocrine therapy; MIBC, muscle invasive bladder cancer; SG, sacituzumab govitecan; EV, enfortumab vedotin; TV, tisotumab vedotin; CT, chemotherapy; IO, immunotherapy; B7-H3, B7 homolog 3 protein; ErbB, epidermal growth factor receptor; GM-CSF, granulocyte–macrophage colony-stimulating factor; mEC, metastatic endometrial cancer; ATR, ataxia telangiectasia and RAD3-related; TROP2, trophoblast cell surface antigen 2; TF, tissue factor; mBC, metastatic breast cancer; mUC, metastatic urothelial cancer; FRα, folate receptor alpha; mNSCLC, metastatic non-small cell lung cancer; FGFR, fibroblast growth factor receptor; TKI, tyrosine kinase inhibitor; MET, cellular-mesenchymal to epithelial transition factor; mCRPC, metastatic castrate resistant prostate cancer; BEV, Bevacizumab; DART, dual-affinity re-targeting antibody, mAb, monoclonal antibody; IgG, immunoglobulin G; NA, not applicable

### Mechanism of ADCs combined with targeted therapy

The combined effects of molecular-targeted drugs (including antibodies) and ADCs synergistically involve multiple mechanisms, such as improving intratumoral drug delivery by targeting tumor blood vessels, regulating tumor cell surface antigen expression, overcoming intratumoral heterogeneity and tumor drug resistance, and synthetic lethality.

### Enhanced cellular uptake and anti-tumor activity

The macromolecular size of ADCs limits extravasation and leads to impaired distribution of ADCs in tumor tissues, ultimately resulting in unsatisfactory efficacy [88]. Two types of barriers affecting ADC delivery have been identified: the blood-tumor barrier, a physical barrier, and the binding-site barrier (BSB), a biological one. Within the blood-tumor barrier microenvironment, blood vessels present in solid tumors are composed of many immature and disorganized vessels, resulting in poor blood flow and hypoxia [89]. Meanwhile, high interstitial oncotic pressure collapses the tumor blood vessels, thus limiting the convective transport of ADCs from the blood into the tumor interstitial fluid [90]. The primary method for altering the tumor vasculature involves modulating angiogenesis and vessel porosity using agents such as anti-vascular endothelial growth factor antibodies such as bevacizumab. Jose F Ponte et al. showed that co-treatment with mirvetuximab soravtansine (an FRα-targeting ADC) and bevacizumab induced swift disruption of tumor microvasculature and extensive necrosis, and improved the efficacy in platinum-resistant epithelial ovarian cancer (EOC) models, emphasizing the superior bioactivity profile of the combination [9]. Another preclinical study showed that the combination of anetumab ravtansine (a mesothelin-targeting ADC) with bevacizumab improved the anti-tumor activity in ST081 and OVCAR-3 human ovarian cancer models [8]. Two studies that investigated the safety and efficacy of mirvetuximab soravtansine in combination with bevacizumab in treating advanced ovarian cancer showed that co-treatment with mirvetuximab soravtansine (6 mg/kg adjusted ideal body weight) and bevacizumab (15 mg/kg), administered intravenously once every three weeks, was well tolerated and presented encouraging efficacy in patients with recurrent epithelial ovarian, fallopian tube, or primary peritoneal cancers [11, 91]. However, there is not just one opinion when it comes to ADC combined with anti-angiogenesis therapy [92–95]. Arjaans et al. reported that the normalization of tumor blood vessels triggered by bevacizumab hampers antibody uptake [92]. The timeframe spanning from normalization to excessive pruning is dependent on both the dose of the anti-angiogenic agent and the duration following administration, which has proven challenging [93]. Importantly, grade 1–2 pneumonitis was detected in six patients (9%) when bevacizumab was introduced alongside mirvetuximab soravtansine, whereas no instances of pneumonitis were observed with the single use of mirvetuximab soravtansine. Therefore, there is an urgent need to rigorously design preclinical and clinical trials to explore the underlying mechanisms of this combination therapy-induced pneumonitis. Co-treatment with ADCs and bevacizumab in a non-clinical trial setting must be performed with caution because of a possible reduction in tumoral accumulation of ADCs that may be caused by bevacizumab.

The BSB is a biological barrier in tumor vasculature regions, which constrains the efficacy of high-affinity antibodies because of the successful binding of antibodies to cellular antigens at the point of extravasation, resulting in antibody sequestration and suboptimal tumor exposure [96]. Many factors, including elevated antigen expression and rapid antigen internalization combined with sluggish tumor uptake and slow interstitial diffusion of therapeutic antibodies, result in poor antibody penetration into the tumor. Transient competitive inhibition, which improves antibody distribution in solid tumors, is one strategy for overcoming BSB. The combined utilization of T-DM1 and pertuzumab showed synergistic activity in cell culture models and had an acceptable safety profile in phase Ib and II studies [97, 98]. Bordeau et al. [99] found that the co-administration of an anti-trastuzumab single domain antibody (1HE) with trastuzumab significantly increased both the penetration of trastuzumab from the vasculature and the percentage of tumor area that stained positive for trastuzumab. 1HE co-administered with a single dose of T-DM1 to NCI-N87 xenograft-bearing mice significantly enhanced T-DM1 efficacy and increased the median survival. However, results from multiple phase III clinical trials (MARIANNE, KRISTINE, and KAITLIN) have shown that the T-DM1 combined with pertuzumab (T-DM1 + P) regimen reduced grade ≥ 3 adverse events and ensured a better quality of life and the this regimen resulted in a higher chance of event-free survival and invasive disease-free survival than regimens of chemotherapy combined with trastuzumab and pertuzumab [72, 100–102]. However, Although monoclonal antibody combined with ADCs could overcome BSB, improve the distribution and anti-tumor efficacy of ADCs in tumor cells, and reduce toxicity, ADC is still not a replacement for standard chemotherapy.

In summary, the dose and time window of ADCs combined with anti-angiogenesis therapy should be further explored in future studies. The establishment of a reasonable model is crucial. Current data show that naked antibodies combined with ADC can overcome BSB to increase tumor penetration and anti-tumor tumor effects; however, they are still unable to replace traditional chemotherapy in clinical settings. 

### Upregulation of surface antigens and overcoming intratumor heterogeneity and drug resistance

Intratumor heterogeneity is a key factor contributing to therapeutic failure. Furthermore, the appearance of compensatory pathways in tumor therapy is one of the mechanisms of drug resistance, which is frequently accompanied by the downregulation of surface antigens [103]. Tumor heterogeneity and drug resistance are major challenges in cancer treatment and research. The different sites of action of monoclonal antibodies and TKI make combination therapy a potential strategy for overcoming these difficulties. The addition of a TKI to a combinational target blockade may provide greater selectivity, with a potentially improved therapeutic index.

Data on TKI that can overcome ADC resistance are scarce. Recently, a preclinical study on T-DM1 resistance was reported. PLK1, a key cell cycle regulator, was upregulated in both acquired and primary T-DM1 resistance models. And inhibition of PLK1 using volasertib led to T-DM1 re-sensitization both in vitro and in vivo [104]. ADCs may also be effective companions for modulating resistance mechanisms of targeted drugs [105–107]. Patients with NSCLC frequently develop acquired drug resistance to EGFR TKIs [108]. HER3 is a unique pseudokinase member of the ERBB family. It dimerizes with other ERBB family members (EGFR and HER2) and is frequently overexpressed in EGFR-mutant NSCLC [109]. Haikala et al. reported that EGFR inhibition by osimertinib leads to increased HER3 membrane expression and promotes HER3-DXd ADC internalization and efficacy, supporting the clinical development of an EGFR inhibitor/HER3-DXd combination in EGFR-mutant lung cancer preclinically [106]. Another example is the co-administration of the EGFR-TKI osimertinib and T-DM1, which contributed to an synergistic anti-tumor effect, where T-DM1 was able to delay or overcome osimertinib resistance in EGFR-mutant NSCLC models [105]. In melanomas, AXL-high cells are resistant to MAPK pathway inhibitors, whereas AXL-low cells are sensitive to them. Heterogeneous tumors show partial therapeutic responses, allowing for the emergence of drug-resistant clones that often express high levels of the receptor tyrosine kinase AXL [110]. Boshuizen et al. [111] found that AXL-107-MMAE and MAPK pathway inhibitors cooperatively inhibited tumor growth by eliminating distinct populations in heterogeneous melanoma cell pools in a preclinical study. Furthermore, the BRAF/MEK inhibitors potentiated the efficacy of AXL-107-MMAE by inducing AXL transcription [111]. In acute myeloid leukemia, a preclinical study found a promising and potent anti-leukemic strategy involving the co-administration of midostaurin (a TKI that inhibits the FLT3 pathway) and a novel FLT3-targeting ADC [112]. The mechanisms behind TKI inducing drug resistance and regulating surface antibody expression are complicated and vary with different drugs. HER2-targeting TKIs (lapatinib, neratinib, tucatinib, and poziotinib) have been shown to increase the efficacy of T-DM1. However, while lapatinib enhances HER2 abundance via robust transcriptional upregulation and reduced ubiquitination, neratinib downregulates surface HER2 abundance by stimulating internalization and endocytosis. The effectiveness of tucatinib on cell surface HER2 is still intricate, and poziotinib upregulates the exon 20 mutant, but not wild-type HER2, suggesting synergistic mechanisms independent of HER2 surface density [21, 113–119].

Several clinical trials have explored the use of TKI in combination with ADCs. The TEAL study showed that employing a combination of T-DM1, lapatinib, and nab-paclitaxel for the neoadjuvant treatment of patients with HER2-positive breast cancer yielded improved responses compared to the standard paclitaxel, trastuzumab, and pertuzumab combination, which was accentuated in the traditionally challenging hormone receptor-positive subset [21]. The combination regimen of T-DM1/T-DXd and tucatinib for advanced breast cancer progression with prior taxane and trastuzumab showed acceptable toxicity and preliminary anti-tumor activity in patients with ERBB2/HER2-positive metastatic breast cancer with and without brain metastases [74, 79, 120]. Moreover, phase III trials testing T-DM1 or T-DXd with tucatinib (HER2CLIMB-02 and HER2CLIMB-04) are ongoing [79, 120], and we look forward to their deterministic results.

### Synthetic lethality and combined targeting

Synthetic lethality is a promising and clinically effective therapeutic strategy for tumors with defects in DNA homologous recombination repair pathways. Given the recent focus on DNA damage response (DDR) pathways in cancer therapy, several DDR proteins, including ATR, ATM, DNA-PK, CHK1, CHK2, Wee1, and PARP, have been extensively explored as promising synthetic lethality targets for anticancer drug development [121–123]. While PARP inhibitors first achieved clinical approval in 2014, inhibitors targeting other DDR proteins are currently under intense clinical investigation.

A subset of ADCs incorporates topoisomerase I (TOPO 1) inhibitors as payloads. For instance, TOPO 1, an enzyme, initiates the cleavage of one strand of double-stranded DNA, resulting in partial unwinding and subsequent reannealing of the strand to relieve tension. Camptothecin and its derivatives bind to the TOPO 1/DNA complex and prevent proper reannealing. This disruption can lead to cell death owing to the accumulation of partially cleaved DNA. SN38, a semi-synthetic derivative of camptothecin, is the active component of irinotecan and has been used in sacituzumab govitecan, a clinically approved TROP2-targeting ADC [124]. Another camptothecin derivative, DXd, is a derivative of exatecan that is approximately 10 times more potent than SN38, with an IC50 value of 310 nM. This potent compound is used as a payload in HER2-targeting (DS-8201a) and TROP2-targeting (DS1062) ADCs.

PARP-1, the most abundant member of the PARP protein family, has been observed to co-localizes with Topo I throughout the cell cycle. However, when DNA damage occurs, PARP-1 dissociates from Topo I, leading to decreased enzymatic activity [125]. Combining a TOPO I inhibitor with a PARP inhibitor (PARPi) results in the accumulation of double-stranded DNA breaks (DSBs) by retarding the homologous recombination repair pathways that effectively and precisely repair DNA damage. This DSB accumulation ultimately triggers apoptosis and cell death. In addition, in cells lacking functional BRCA1/2 genes or those deficient in the homologous recombination repair mechanism, an alternative but less precise DNA damage repair pathway known as non-homologous end-joining emerges. This more error-prone pathway further compromises cells toward irreparable DNA damage and apoptosis [126]. Recent research has also shown that combining CPT-11 (the prodrug of SN-38) and PARPi achieved synergistic inhibition in both BRCA1 wild-type and BRCA1 mutant triple-negative breast cancer (TNBC) cell lines in vitro [127]. Concurrently, sacituzumab govitecan combined with olaparib, rucaparib, or talazoparib also synergistically inhibited tumor cell growth and increased DSBs in HCC1806 TNBC tumors harboring mutations in the BRCA1/2 genes, as well as in those with wild-type counterparts [128].

### Safety profile of ADCs combined with targeted therapies

Among the 15 types of ADC currently approved, the combination of T-DM1 and targeted agents has the greatest evidence of efficacy and safety. A phase Ib trial evaluated the combination of T-DM1 and the HER2 TKI, tucatinib. Although this combination is well-tolerated, it is associated with frequent gastrointestinal and hepatic toxicities. In particular, 37% of the patients experienced at least one clinically significant adverse event, and 56% required discontinuation of tucatinib [74]. More information is expected from the ongoing phase III HER2CLIMB-02 trial evaluating T-DM1 with tucatinib. Compared with T-DM1 alone, these results may provide more insight into the additional toxicity associated with this combination. Another Ib study evaluated the combination of intermittent inhibition of T-DM1 and CDK4/6 using riboflavin; however, no DLTs were observed. However, ribociclib dose reductions were required in 58% of the patients due to thrombocytopenia or neutropenia, and one patient experienced grade 2 QTcF prolongation [129]. In a phase Ib study involving patients with metastatic TNBC, sacituzumab govitecan was administered in combination with talazoparib, a PARP inhibitor. This study demonstrated several DLTs primarily caused by severe myelosuppression, as described in the initial study results. In particular, the majority of enrolled patients experienced febrile neutropenia [83]. Finally, a phase Ib study evaluated the effects of adding bevacizumab to mirvetuximab soravtansine. This study included patients with platinum-resistant ovarian cancer [11]. The combination resulted in a toxicity profile similar to that of ADC alone [130]. However, it is worth noting that grade 1–2 pneumonitis was observed in six patients (9%) with the addition of bevacizumab. This was in contrast to the absence of pneumonitis when mirvetuximab soravtansine was administered alone.

In summary, although we observed promising results from combining TKIs and ADCs in preclinical models and clinical trials, the underlying molecular interplay is still far from being completely understood. A better mechanistic understanding is helpful for the selection of drug combinations and the management of potential side effects.

## ADCs combined with immunotherapy

Accumulating evidence suggests that ADCs are sensitive to the effectiveness of immunotherapeutic agents [131]. Combining immunotherapy with ADCs is a current trend in clinical practice, with a number of preclinical studies and initial findings from early-stage clinical trials showing improved anti-tumor effects [131]. The clinical trials are listed in Table 6. We still await the outcomes of large-cohort randomized phase III clinical trials to demonstrate the determinant evidence of this combination’s efficacy compared with that of conventional treatments.

**Table 6:** Summary of clinical trials investigating the combination of ADCs and immunotherapy

Target	NCT number	Other names	Drug	Partner drugs	Partner drug category (target)	Phase	Start	Treatment setting	Efficacy
HER2	NCT02605915 [139]	NA	T-DM1	Atezolizumab	IO	Ib	2015	mBC	Positive
	NCT02924883 [140]	KATE2	T-DM1	Atezolizumab	IO	II	2016	mBC	Negative
	NCT03364348 (completed)	NA	T-DM1	Utomilumab	4-1BB	IB	2017	mBC	NA
	NCT03032107 [141]	NA	T-DM1	Pembrolizumab	IO	Ib	2017	mBC	NA
	NCT03523572 [77]	DS8201-A-U105	T-DXd	Nivolumab	IO	Ib	2018	mBC & mUC	Positive
	NCT04042701 [78]	NA	T-DXd	Pembrolizumab	IO	Ib	2019	mBC & mNSCLC	NA
	NCT04379596 [142]	DESTINY-Gastric03	T-DXd	5-FU	CT	Ib/II	2020	mGC	NA
				Durvalumab	IO				
				Capecitabine	CT				
				CAPOX	CT				
				5-FU and Durvalumab	CT + IO				
				Capecitabine, and Durvalumab	CT + IO				
				5-FU or Capecitabine, and Cisplatin or Oxaliplatin	CT				
				Chemotherapy (FP) and Pembrolizumab	CT + IO				
				Chemotherapy and BSP (MEDI5752)	CT + IO				
				5-FU or Capecitabine, and Pembrolizumab	CT + IO				
				Pembrolizumab	IO				
	NCT04556773 (active, not recruiting)	DB-08	T-DXd	Durvalumab	IO	Ib	2020	mBC	NA
				Capecitabine	CT				
				Capivasertib	CT				
				Durvalumab + Paclitaxel	CT + IO				
				Anastrozole	ET				
				Fulvestrant	ET				
	NCT04538742 [81]	DB-07	T-DXd	Durvalumab	IO	Ib/2	2020	mBC	NA
				Tucatinib	HER2 TKI				
				Pertuzumab	HER2 mAb				
				Paclitaxel	CT				
				Durvalumab + Paclitaxel	CT + IO				
	NCT05480384 (recruiting)	BrUOG 413	T-DXd	Nivolumab	IO	II	2022	Adjuvant, esophagogastric adenocarcinoma	NA
	NCT04264936 [80]	RC48-C014	RC48	Toripalimab	IO	Ib/II	2020	mUC	Positive
	NCT04460456 [143]	NA	SBT6050	Pembrolizumab	IO	I	2020	Advanced solid tumors	Positive
				Cemiplimab	IO				NA
	NCT05113459 (not yet recruiting)	RC48-C018	RC48	Sintilimab and Capecitabine	CT + IO	II	2021	Neoadjuvant, GC	NA
	NCT04879329 [144]	RC48G001	RC48	Pembrolizumab	IO	II	2021	mUC	NA
	NCT05016973 (not yet recruiting)	NA	RC48	Triplizumab	IO	II	2021	Neoadjuvant, MIBC	NA
	NCT04873362 [145]	Astefania	T-DM1	Atezolizumab	IO	III	2021	Adjuvant, BC	NA
	NCT04740918 [146]	KATE3	T-DM1	Atezolizumab	IO	III	2021	mBC	NA
	NCT05488353 (not yet recruiting)	NA	RC48	Penpulimab Injection	IO		2022	Neoadjuvant, bladder urothelial carcinoma	NA
	NCT05495724 (recruiting)	NA	RC48	Tislelizumab	IO	II	2022	High-Risk Non-Muscle Invasive Bladder Cancer	NA
	NCT05493683 (recruiting)	NA	RC48	Tislelizumab	IO	II	2022	mCRC	NA
	NCT05333809 (not yet recruiting)	NA	RC48	Pembrolizumab	IO	II	2022	mCRC	NA
	NCT05313906 (not yet recruiting)	NA	RC48	AK105 + Cisplatin	CT + IO	II	2022	mGC	NA
	NCT05417230 (not yet recruiting)	NA	RC48	Envafolimab	IO	II	2022	mBTC	NA
	NCT05115500 (not yet recruiting)	PRaG3.0	RC48	Hypofractionated RT, PD-1/PD-L1 inhibitor sequential GM-CSF and IL-2	IO + RT	II	2022	Advanced solid tumors	NA
	NCT05297552 (recruiting)	NA	RC48	Toripalimab	IO	II	2022		NA
	NCT05302284 (recruiting)	NA	RC48	Toripalimab	IO	III	2022	mUC	NA
	NCT05320588 (recruiting)	NA	BIO-106	Pembrolizumab	IO	I/II	2022	Advanced solid tumors	NA
	NCT05514158 (recruiting)	NA	RC48	Chemotherapy (Capecitabine or 5-FU) + Nivolumab	CT + IO	I	2022	mGC	NA
				RC98	IO				
	NCT05979740 (recruiting)	NA	RC48	Toripalimab + RT	IO + RT	II	2023	MIBC	NA
TROP2	NCT03742102 [147]	BEGONIA	T-DXd	Durvalumab	IO	IB/II	2018	mBC	Positive
			Dato-DXd	Durvalumab	IO				NA
	NCT03337698 [148]	Morpheus Lung	SG	Atezolizumab	IO	Ib/II	2017	mNSCLC	
	NCT03424005 (active, not recruiting)	Morpheus-panBC	SG	Atezolizumab	IO	Ib/II	2018	mBC	NA
	NCT03971409 (recruiting)	InCITe	SG	Avelumab	IO	II	2019	mBC	NA
	NCT03869190 [149]	MORPHEUS-UC	SG	Atezolizumab	IO	Ib/II	2019	mUC	NA
	NCT04434040 [150]	ASPRIA	SG	Atezolizumab	IO	II	2020	Adjuvant, BC	NA
	NCT04468061 [151]	NA	SG	Pembrolizumab	IO	II	2020	mBC	NA
	NCT04448886 [152]	NA	SG	Pembrolizumab	IO	II	2020	mBC	NA
	NCT04381832 [153]	NA	SG	Etrumadenant + Zimberelimab	Dual adenosine receptor antagonist + IO	I/II	2020	mCRPC	Positive
	NCT04863885 [154]	NA	SG	IPI-NIVO	IO	I/II	2021	mUC	Positive
	NCT05382286 (recruiting)	ASCENT-04	SG	Pembrolizumab	IO	III	2022	mBC	NA
	NCT05186974 [155]	EVOKE-02	SG	Pembrolizumab	IO	II	2022	mNSCLC	NA
				Pembrolizumab and a platinum agent (Carboplatin or Cisplatin)	CT + IO				
	NCT05327530 [156]	JAVELIN Bladder Medley	SG	Avelumab	IO	II	2022	mUC	NA
	NCT05687266 (recruiting)	AVANZAR	Dato-DXd	Durvalumab + Carboplatin	CT + IO	III	2022	mNSCLC	NA
	NCT05489211 [157]	TROPION-PanTumor03	Dato-Dxd	Durvalumab	IO	II	2022	Advanced solid tumors	NA
				Durvalumab + AZD5305	IO + PARP1 inhibitor				
	NCT04526691 [158]	TROPION-Lung02	Dato-Dxd	Pembrolizumab	Carboplatin/ Cisplatin	I	2020	Advanced or metastatic NSCLC	Positive
	NCT04612751 [159]	TROPION-Lung04	Dato-Dxd	Durvalumab AZD2936 MEDI5752	Carboplatin	Ib	2020	Advanced or metastatic NSCLC	NA
	NCT05941507 (not yet recruiting)	NA	LCB84	Anti-PD-1	IO	I/II	2023	Advanced solid tumors	NA
Nectin-4	NCT03924895 [160]	KEYNOTE-905/EV-303	EV	Pembrolizumab	IO	III	2019	Perioperative, MIBC	NA
	NCT04223856 [161]	EV-302	EV	Pembrolizumab	IO	III	2020	mUC	Positive
				Pembrolizumab + Cisplatin or Carboplatin	CT + IO				NA
	NCT04960709 [162]	VOLGA	EV	Durvalumab + Tremelimumab	IO	III	2021	Perioperative, MIBC	NA
				Durvalumab	IO				
	NCT04700124 [163]	MK-3475-B15/ KEYNOTE-B15 / EV-304	EV	Pembrolizumab	IO	III	2021	Perioperative, MIBC	NA
	NCT05239624 (recruiting)	EV-ECLIPSE	EV	Pembrolizumab	IO	II	2022	Neoadjuvant, UC	NA
	NCT05756569 (not yet recruiting)	NA	EV	Pembrolizumab	IO	II	2023	mUC	NA
	NCT05775471 (not yet recruiting)	NA	EV	Pembrolizumab	IO	II	2023	Perioperative, High-risk upper tract urothelial cancer	NA
EGFR	NCT04305795 (active, not recruiting)	NA	ASP-1929	Pembrolizumab	IO	I/II	2020	Advanced solid tumors	NA
				Cemiplimab	IO				
EGFR	NCT05265013 (active, not recruiting)	NA	ASP-1929	Pembrolizumab	IO	II	2022	Locoregional recurrent SCCHNC, with or without metastases, not amenable to curative local treatment	NA
ROR2	NCT03504488 (recruiting)	NA	CAB-ROR2-ADC	PD-1 inhibitor	IO	I/II	2018	Advanced solid tumors	NA
FRα	NCT02606305 [28]	NA	Elahere	Pembrolizumab	IO	Ib/II	2022	High-grade epithelial ovarian, primary peritoneal, or fallopian tube cancers	NA
	NCT03835819 [164]	NA	Elahere	Pembrolizumab	IO	II	2019	mEC	NA
AXL	NCT03425279 [165]	NA	CAB-AXL-ADC	PD-1 inhibitor	IO	I/II	2018	Advanced, refractory sarcoma	NA
AXL	NCT04681131 (recruiting)	NA	CAB-AXL-ADC	PD-1 inhibitor	IO	II	2021	mNSCLC	NA

T-DM1, Ado-trastuzumab emtansine; T-DXd, fam-trastuzumab deruxtecan; HER2, human epidermal growth factor receptor 2; HER3, human epidermal growth factor receptor 3; mBC, metastatic breast cancer; mUC, metastatic urothelial cancer; ET, endocrine therapy; MIBC, muscle invasive bladder cancer; SG, sacituzumab govitecan; EV, enfortumab vedotin; TV, tisotumab vedotin; CT, chemotherapy; IO, immunotherapy; B7-H3,B7 homolog 3 protein; ErbB, epidermal growth factor receptor; GM-CSF, granulocyte–macrophage colony-stimulating factor; mEC, metastatic endometrial cancer; ATR, ataxia telangiectasia and RAD3-related; TROP2, trophoblast cell surface antigen 2; TF, tissue factor; FRα, folate receptor alpha; mNSCLC, metastatic non-small cell lung cancer; FGFR, fibroblast growth factor receptor; TKI, tyrosine kinase inhibitor; MET, cellular-mesenchymal to epithelial transition factor; mCRPC, metastatic castrate resistant prostate cancer; Dato-Dxd, datopotamab deruxtecan; IPI-NIVO, nivolumab (Nivo) and ipilimumab (Ipi); SCCHNC, squamous cell carcinoma of the head and neck; 4-1BB, T cell co-stimulatory receptor agonist; MIBC, muscle invasive bladder cancer; NA, not applicable

### Mechanism of ADCs combined with immunotherapy

The underlying mechanisms are diverse and encompass Fc-mediated effector functions, initiation of immunogenic cell death (ICD), maturation of dendritic cells (DCs), enhancement of T cell infiltration, reinforcement of immunological memory, and expression of immunomodulatory proteins such as programmed death ligand 1 (PD-L1) and major histocompatibility complex (MHC) [132–136]. Multiple studies have revealed that ADCs exert a stronger effect in immunocompetent animal models than in immunodeficient models, indicating their significant immunomodulatory capabilities [137, 138]. These findings provide a basis for devising clinical trials that incorporate low doses of ADCs as immunostimulants to improve the efficacy of immunotherapy without causing adverse effects.

### Fc-mediated effector functions

In the design of an ADC, the antibody component plays a multifaceted role, instead of solely delivering cytotoxic agents to cancer cells. Its unique Y-shaped structure has many additional functions, including regulation of innate immune responses. While the antigen-binding fragments of an antibody are responsible for recognizing the target antigen and determining its specificity, the crystallizable fragment (Fc) interacts with immune cells and regulates the duration of the antibody’s circulation in the bloodstream. The Fc region, in fact, plays key roles in several vital functions, including antibody-dependent cell-mediated cytotoxicity (ADCC), antibody-dependent cell-mediated phagocytosis, and complement-dependent cytotoxicity. The effectiveness of the initial two actions relies on Fcγ receptors (FcγRs), which are present on natural killer cells, macrophages, and various other immune cells. Conversely, complement-dependent cytotoxicity is triggered by C1q protein.

The IgG antibody family consists of IgG1, IgG2, IgG3, and IgG4 subclasses, each of which exert different effects on factors such as antibody solubility, half-life, interaction with the C1q protein, and binding strength to FcγRs. The IgG1 subclass of antibodies is most commonly used in the 15 clinically approved types of ADCs [166]. The reason for selecting IgG1 as the backbone of ADC is because it has a long half-life of approximately 21 days, similar to IgG2 and IgG4, but is characterized by its enhanced ability to activate the complement system and bind to FcγRs. In contrast, IgG2 and IgG4 have limited efficacy in triggering effector functions, and are used strategically in antibody design when eliciting an immune response is not the primary goal [167]. In contrast, IgG3 is the most immunogenic subclass capable of eliciting an immune response. However, these antibodies are typically bypassed in the design of ADCs because of their short half-lives (approximately seven days).

In preclinical models, both T-DXd and T-DM1 exhibit the ability to maintain functions inherent to unconjugated trastuzumab, including triggering ADCC associated with the IgG1 isotype [137, 168]. In addition to these therapeutic benefits, Fc-mediated effector functions give rise to undesirable side effects. For instance, T-DM1 is internalized by megakaryocytes through its interaction with FcγRIIA, which could potentially lead to the development of thrombocytopenia, a known side effect of this agent [169].

Modulating the ability of an ADC to engage the immune system may involve engineering the Fc region. One approach is to produce afucosylated IgGs, which enhance ADCC by increasing the binding affinity for FcγRIIIa [170, 171]. Conversely, the Fc region can be modified by introducing mutations that impact effector functions, yielding what is known as "Fc silent antibodies" [138]. MEDI4276, for instance, employs this strategy with three mutations in its Fc domain to curtail FcγR binding, aiming to minimize thrombocytopenia as observed with T-DM1 [172].

Another approach that involves the interaction between Fc and FcγRs takes place in the TME, in which the tumor-associated macrophages (TAMs) constitute a significant proportion. In preclinical models, non-targeted ADCs have been shown to be effectively engulfed by TAMs. Through the engagement of FcγRs, TAMs internalize and process these ADCs, resulting in the release of the cytotoxic payloads within the TME. This results in the killing of neighboring tumor cells which is called “bystander effect”. This mechanism may enhance the efficacy of ADCs against tumors that exhibit heterogeneity or low levels of target antigens [173].

However, it is important to recognize that this mechanism of antigen-independent ADC uptake into non-malignant cells within the TME could exacerbate the toxicity of ADCs. For instance, it could potentially lead to more rapid clearance of ADCs and reduced overall efficacy. Another theoretical concern related to the release of cytotoxic payload within the TME and the subsequent bystander effect is the potential destruction of local T cells, which can negatively affect the efficacy of immune checkpoint inhibitors (ICIs).

In summary, while the interaction of Fc and FcγRs between ADCs and the TME represents a potential avenue for enhancing ADC activity against tumors with challenging characteristics, careful consideration must be given to balance efficacy with potential drawbacks such as increased toxicity and interference with immune checkpoint inhibition.

### Immunogenic cell death

Based on the initial stimulus, cancer cell death can either activate the immune system (immunogenic) or go unnoticed by it (non-immunogenic). ICD is a regulatory process characterized by the induction of stress within the endoplasmic reticulum and cellular structures. This process is accompanied by changes in the cell surface composition and the subsequent release of soluble mediators, which follow a precise spatiotemporal sequence and ultimately lead to cell death [174, 175].

The ability of a drug to induce ICD and establish an immunological memory is often predicted by its ability to induce damage-associated molecular patterns (DAMPs) in vitro [176, 177]. A wide array of anticancer therapeutics, including traditional chemotherapy, radiotherapy, and targeted anticancer agents, has demonstrated the potential to induce DAMPs [178–180]. Only a small fraction (< 10%) of all chemotherapeutic agents, such as anthracyclines [181, 182] and oxaliplatin [183], are classified as ICD-inducing drugs. The majority of cytotoxic payloads employed in ADCs exhibit the ability to activate immune cells both in the laboratory and in living organisms, which not only improves their anti-tumor tumor efficacy, but also synergistically enhances the effect of ICIs in preclinical models [184].

In mouse models, ADCs with payloads such as maytansine, pyrrolobenzodiazepine, and tubulysin have shown the ability to induce ICD [135] trigger immune modulation, and establish immune memory. These ADCs not only exhibit potent cytotoxicity but also synergize with various ICIs. Notably, these ADCs exhibited significantly greater anti-tumor activity in immunocompetent mice than in immunocompromised mice, highlighting the role of the immune system in their efficacy. Similarly, a newly developed anti-HER2 ADC containing a potent anthracycline derivative payload (T-PNU) increased DAMP expression and enhanced efficacy when combined with an anti-PD-1 drug in a breast cancer model that developed resistance to other HER2-targeted therapies. Notably, the efficacy of T-PNU was significantly reduced when CD8 + T cells were depleted, confirming the critical role of the adaptive immune system in regulating the anticancer activity of T-PNU. In addition, T-PNU appeared to promote the formation of an immunological memory in tumor-bearing animals, resulting in protection against tumor rechallenge [132]. Such an ICD-induced intrinsic inflammatory response has also been observed with brentuximab vedotin [185], ladiratuzumab vedotin [186], and enapotamab vedotin [136], which are three ADCs that share the same MMAE payload. This unique property further enhances the efficacy of ICIs.

### Direct activation and maturation of dendritic cells

Mature DCs play a pivotal role in tumor immunity by acting as antigen-presenting cells capable of activating anti-tumor T cell responses through the MHC class II complex [187]. However, cancer cells often develop immunosuppression by inhibiting DC maturation or causing them to become dysfunctional, ultimately resulting in immune evasion [188]. Overcoming these barriers is essential for improving immunotherapy outcomes in clinical practice.

Previous research has shown that compounds that destabilize microtubules are capable of inducing the phenotypic and functional maturation of DCs, which was not observed with microtubule-stabilizing compounds such as taxanes [189]. This phenomenon appears to be a common feature of this class of compounds, indicating their potential use as "immunostimulatory" agents. This immunostimulatory effect was first reported in vinblastine [190] and subsequently in many other microtubule-disrupting agents, such as maytansinoids (e.g., ansamitocin P3 and its synthetic derivative DM1) and dolastatins (from which auristatins are derived), which are frequently used as payloads in ADCs [133, 191]. In preclinical models, these payloads have been demonstrated to directly trigger DC activation and maturation. Importantly, these potent immunoregulatory effects were observed even without cancer cell death, indicating an independent binary mode of action. The complete therapeutic efficacy of these ADCs includes payload cytotoxicity and immunoregulatory functions; the latter strongly depends on an intact host immune system, which is significantly diminished in immunocompromised mouse models.

Preclinical studies combining tubule inhibitor-based ADCs with ICIs have confirmed that these two types of treatment modalities can work synergistically to increase therapeutic efficacy rather than have additive effects [134, 191]. In cases where tumors responded completely to the combination treatment, mice showed protection upon rechallenge with the same tumor, indicating the successful establishment of immunological memory. Notably, an analysis of paired samples from 28 patients with breast cancer who underwent short-term preoperative treatment with T-DM1 as part of the WSG-ADAPT protocol sub-trial revealed significant increases in the number and density of tumor-infiltrating T cells [168]. There is evidence that topoisomerase I inhibitors can also act as immunomodulators by activating DCs [192], as exemplified by T-DXd, an HER2-targeted ADC that carries the exatecan derivative DXd, a topoisomerase I inhibitor, as payload. T-DXd has been found to significantly increase the presence of tumor-infiltrating DCs and the expression of markers indicative of maturation and activation, leading to an increase in tumor-infiltrating CD8 + T cells, along with increased expression of PD-L1 and MHC class I on tumor cells. Notably, in a CT26-HER2 tumor model, the combination of T-DXd with an anti-PD-1 agent proved to be more effective than either treatment alone, possibly because of the immunomodulatory changes induced by T-DXd [134]. It is worth noting that the T-DXd payload possesses a tenfold more potent topoisomerase I inhibitor activity compared to SN-38, which may contribute to a more robust immunologic effect compared to other agents in the same class [168].

### Combining ADCs and ICIs

Currently, HER2-targeted ADCs are under intense clinical investigation for their synergistic effects in combination with ICIs; however, many trials are still ongoing, and determinant evidence is very limited [137, 193, 194]. The only published randomized trial evaluating the combination of an ADC and ICI is the KATE2 trial. This study evaluated the efficacy of T-DM1 plus atezolizumab and compared it with T-DM1 plus placebo. The study was conducted in patients who had previously been treated for HER2-positive breast cancer and, disappointingly, the combination therapy did not result in a statistically significant improvement in PFS in the overall patient population, with a median PFS of 8.2 months in the combination arm and 6.2 months in the control arm (*P* = 0.33). However, a trend suggesting potential benefit in a specific subset of patients with positive PD-L1 expression was observed, where median PFS was 8.5 months for the combination arm and 4.1 months for the control arm (*P* = 0.099). This indicated that adding an ICI to HER2-targeted treatment for HER2 + breast cancer may be particularly beneficial in the PD-L1-positive subset [77, 195–204]. Most cancer patients enrolled in published studies had not previously received treatment with an ICI. Thus, we are uncertain about the synergistic benefits that ADC may provide when combined with ICIs in tumor types that are known to respond well to immunotherapy. However, currently available clinical data suggest significantly improved response rates, as shown by a comparison with the historical efficacy results achieved with standalone immunotherapy in these specific tumor types [132, 133, 135, 136, 184–190].

After witnessing remarkable improvements in efficacy in specific cancer contexts, certain combinations will likely be adopted as new standards of care, potentially replacing traditional cytotoxic treatment approaches. To illustrate this theory, the combination of enfortumab vedotin, an ADC targeting nectin 4, and pembrolizumab has been evaluated as a first-line treatment option for individuals diagnosed with locally advanced or metastatic urothelial cancer (as demonstrated in the EV-103/KEYNOTE-869 study; ClinicalTrials.gov identifier: NCT03288545) [184]. In this patient population, the combination achieved an impressive objective response rate of 73% and extended the PFS to 12.3 months. This result led to a breakthrough designation granted by the US FDA, specifically for patients ineligible for cisplatin-based therapy. Moreover, other cancers, including cervical cancer, triple-negative breast cancer, Hodgkin's lymphoma, and primary mediastinal large B cell lymphoma, have shown encouraging results when treated with a combination of anti-PD-1 antibodies and ADCs targeting specific markers, such as tissue factor, TROP2, and CD30 [205]. This novel strategy of combination therapy holds great promise, particularly for older and frail patients who are at an increased risk of experiencing severe side effects from traditional chemotherapy regimens [187, 188, 190, 206–208]. In the coming months and years, we expect to see more trial results that employ various immunotherapeutic agents to enhance ADC activity.

### Safety profile of ADCs combined with immunotherapy

In the phase III KATE2 trial, a significant increase in adverse events, including one treatment-related death, was observed in the combination arm that combined atezolizumab and T-DM1 in patients with previously treated HER2-positive metastatic breast cancer. The frequencies of clinically significant adverse events (33% vs. 19%) and most adverse reactions, particularly fever (35% vs. 16%, including several hospitalizations), increased after the introduction of atezolizumab [140]. Similarly, randomized data are now available for enfortumab vedotin, with and without pembrolizumab, in 149 patients with advanced-stage urothelial carcinoma. The introduction of pembrolizumab resulted in a higher occurrence of clinically significant (23.7% vs. 15.1%) and fatal (3.9% vs. 2.7%) adverse events, and an overall increase in the incidence of all adverse events. Notably, an increase in serious skin reactions was observed [209]. Nevertheless, the combination received accelerated approval from the FDA in April 2023 for patients with locally advanced or metastatic urothelial carcinoma, particularly those who could not receive cisplatin-based chemotherapy. This approval was based on the efficacy of the combination. However, the lack of a randomized design in most other studies on ADC and ICI combination therapies makes it difficult to reach definitive conclusions regarding the many unanswered questions. To date, there have been no alarming signs of increased toxicity induced by ICIs in combination with T-DXd [77, 210, 211], Dato-DXd [204], or sacituzumab-govitecan [212], all of which have additive toxicity profiles. These safety profiles include similar rates and intensities of ILD, which frequently occurs with DXd-based ADC treatments. Interestingly, the addition of ICIs does not appear to increase the incidence of ILD associated with ADCs [141, 175, 177, 213]. Ongoing randomized phase III trials (NCT05629585, NCT05382286, and NCT05633654) are expected to offer additional insights into this domain. These trials may further clarify the toxicity patterns of treatment approaches that combine ICIs with ADCs beyond T-DM1.

## Conclusions

Monotherapy with ADCs has exhibited transformative anti-tumor efficacy across a broad spectrum of solid and hematological malignancies. The present landscape is characterized by substantial efforts from both the academic and industrial sectors focused on advancing the understanding of ADC combination therapy, which entails the progress of next-generation ADCs by identifying novel tumor targets and clarifying their pharmacological properties.

Notably, the combination of ADCs with chemotherapy or chemoimmunotherapy regimens, excluding ICIs, has yielded demonstrable survival advantages over the established standard regimens in randomized investigations of hematological malignancies. Although the combination of ADCs with ICIs has exhibited encouraging outcomes, as exemplified by the FDA breakthrough designations of enfortumab vedotin and pembrolizumab for cisplatin-ineligible urothelial cancer, the envisioned survival improvements and underlying biological mechanisms for solid tumors remain elusive within the context of randomized controlled trials.

Furthermore, ADC combinations with targeted agents, particularly inhibitors targeting the HER2 and DDR pathways, hold substantial promise, although their potential is contingent upon validation through more mature datasets. The constrained success witnessed thus far in combination therapy with first-generation ADCs (e.g. T-DM1) can be attributed to many factors that encompass the indiscriminate expression of target molecules, resulting in off-tumor side effects on normal tissues, overlapping toxicities, limited efficacy, and unclear procedures conferring resistance. The landscape of ADC-based combination therapies remains dynamic, with current challenges underscoring the complexities of target expression, tumor heterogeneity, and the intricate interplay of therapeutic modalities.

In addition to understanding the pharmacological properties (e.g. DAR and bystander effect) to improve ADC efficacy, there is also a strong need to stratify patients with high response rates and detect relevant predictive biomarker profiles. Exploring preclinical experiments in carefully characterized patient-derived xenograft models and conducting clinical trials in window-of-opportunity contexts could facilitate the identification of promising ADC-based combinations in clinical practice. More strategic methodologies are required to effectively identify suitable ADC-based combination approaches for selected patient cohorts and tumor types. This will not only capitalize on the refinement of ADC design and properties, but also leverage well-informed patient selection strategies to optimize therapeutic outcomes.

## Data Availability

Not applicable.
